# *Cauli*: A Mouse Strain with an Ift140 Mutation That Results in a Skeletal Ciliopathy Modelling Jeune Syndrome

**DOI:** 10.1371/journal.pgen.1003746

**Published:** 2013-08-29

**Authors:** Kerry A. Miller, Casey J. Ah-Cann, Megan F. Welfare, Tiong Y. Tan, Kate Pope, Georgina Caruana, Mary-Louise Freckmann, Ravi Savarirayan, John F. Bertram, Michael S. Dobbie, John F. Bateman, Peter G. Farlie

**Affiliations:** 1 Murdoch Childrens Research Institute, Parkville, Victoria, Australia; 2 Department of Paediatrics, University of Melbourne, Parkville, Victoria, Australia; 3 Victorian Clinical Genetics Service, Royal Children's Hospital, Parkville, Victoria, Australia; 4 Department of Anatomy and Developmental Biology, School of Biomedical Sciences, Monash University, Clayton, Victoria, Australia; 5 Sydney Children's Hospital, Randwick, New South Wales, Australia; 6 The Australian Phenomics Facility, The Australian National University, Canberra, Australian Capital Territory, Australia; 7 Department of Biochemistry and Molecular Biology, University of Melbourne, Parkville, Victoria, Australia; Washington University School of Medicine, United States of America

## Abstract

Cilia are architecturally complex organelles that protrude from the cell membrane and have signalling, sensory and motility functions that are central to normal tissue development and homeostasis. There are two broad categories of cilia; motile and non-motile, or primary, cilia. The central role of primary cilia in health and disease has become prominent in the past decade with the recognition of a number of human syndromes that result from defects in the formation or function of primary cilia. This rapidly growing class of conditions, now known as ciliopathies, impact the development of a diverse range of tissues including the neural axis, craniofacial structures, skeleton, kidneys, eyes and lungs. The broad impact of cilia dysfunction on development reflects the pivotal position of the primary cilia within a signalling nexus involving a growing number of growth factor systems including Hedgehog, Pdgf, Fgf, Hippo, Notch and both canonical Wnt and planar cell polarity. We have identified a novel ENU mutant allele of *Ift140*, which causes a mid-gestation embryonic lethal phenotype in homozygous mutant mice. Mutant embryos exhibit a range of phenotypes including exencephaly and spina bifida, craniofacial dysmorphism, digit anomalies, cardiac anomalies and somite patterning defects. A number of these phenotypes can be attributed to alterations in Hedgehog signalling, although additional signalling systems are also likely to be involved. We also report the identification of a homozygous recessive mutation in IFT140 in a Jeune syndrome patient. This ENU-induced Jeune syndrome model will be useful in delineating the origins of dysmorphology in human ciliopathies.

## Author Summary

Skeletal ciliopathies are an emerging field of human disease in which skeletal birth defects arise due to abnormal communication between cells. This failure in communication arises following mutation in components of the primary cilia, a hair-like structure present on every cell. The skeletal ciliopathies are debilitating and in severe cases lead to death in early infancy. However, the mechanisms by which these malformations come about remains unclear. Mouse models are often used to delineate the causes of human birth defects and we have identified a model that mimics one of these conditions known as Jeune syndrome. It is the first mouse model with a mutation in the *Ift140* gene, and these mice exhibit phenotypes that are often seen in this set of human syndromes. We have complimented this study with the discovery of a patient that presents with Jeune Syndrome resulting from mutation of human *IFT140*. This model will allow us to explore the role of IFT140 and the primary cilia in normal human development and provide insight into the field of human skeletal ciliopathies.

## Introduction

Primary cilia provide highly regulated cellular sensory functions with diverse roles in the development and maintenance of many tissues and organs [1]. Primary cilia protrude from the surface of most eukaryotic cells except fungi and higher plants. The structural support for this arrangement comes from a tubulin-based scaffold known as the axoneme, the assembly and function of which is coordinated through a centriole-derived basal body. The basal body is tethered to the plasma membrane by transition fibres that are believed to form a selective gate or pore which functions to control the movement of proteins between the cytoplasm and cilium [2]. The sensory function of the cilia derives from the deployment of plasma membrane-spanning receptors and channels within the ciliary membrane that provide a link between the extracellular environment and the cytoplasm [1].

Components of the primary cilia have to be transported from their site of synthesis within the cytoplasm to the cilium itself using a microtubule motor-based system known as intraflagellar transport (IFT). The axoneme grows from the distal tip and individual axonemal subunits must be transported along the nascent axoneme by IFT [3]. The IFT complex is simultaneously in close physical association with both the axoneme and the ciliary membrane enabling transport of membrane bound proteins such as growth factor receptors [2]. Given the blind ending structure of the cilia, IFT must proceed in a two-way fashion. The IFT particle is composed of at least two distinct sub-complexes, complex B and complex A, which are specialized for either outbound (anterograde) transport or the return (retrograde) journey respectively [4], [5], [6], [7]. The IFT particles form linear arrays known as IFT trains, which are transported by different motor protein complexes depending on the direction of movement. The anterograde transport complex B uses a kinesin-based motor while the retrograde transport complex A uses a dynein-based motor system [8].

The IFT-A complex consists of at least 6 subunits with a highly stable core sub-complex of three proteins, while the IFT-B complex consists of around 14 subunits with a salt-stable core of 9 [9]. The nature of the interaction between the IFT-A and B complexes and their respective motor protein complexes remains unclear but anterograde transport components become cargo for the retrograde complex and the converse is also true [10], [11]. Thus, mutation of any individual subunit is likely to impact on the function of the entire system. However, each transport complex has specific roles within the cilia, mutations within IFT-B tend to result in short or absent cilia while mutations in IFT-A more typically result in distorted cilia with a distended tip due to accumulation of stranded IFT cargo [12].

Over the past decade our appreciation of the importance of primary cilia function in development and disease has grown rapidly. There are now at least 12 disorders that have been attributed to mutations in cilia proteins and collectively they impact on nearly every organ system in the body [13]. The skeletal ciliopathies are a diverse group of conditions involving congenital malformation of variable skeletal systems including the craniofacial skeleton, ribs and limbs. One example is the short-rib polydactyly (SRP) group which includes at least 6 distinct autosomal recessive conditions including Jeune syndrome [14]. Common features of the SRPs include a small thoracic cage due to a failure of normal rib growth, shortened tubular bones and pelvic defects. In addition, individual conditions present with a diverse range of soft-tissue defects including malformations of the heart, intestine, genitalia, pancreas, liver and polycystic kidneys. There is significant phenotypic overlap between these conditions and recent identification of causative genetic mutations confirms that the difficulty in distinguishing the features of particular SRPs stems from defects common to the structure or function of the primary cilia [15].

The primary cilia are endowed with an array of signal transduction components such as ion channels, receptor tyrosine kinases, Notch receptors and matrix receptors including the Hedgehog signalling components Ptch1, Smo1 and Gli, PDGFRa, IGFR1, EGFR and Tie2 [16]. The function and activity of these sensory components is regulated, at least in part, by controlling their localisation within the cilia and in part by controlling access to downstream signalling components. The co-localisation of multiple signal transduction components within the cilia raises the possibility that the cilia act as a site to coordinate cross talk between signalling systems [16]. Thus, the broad range of phenotypes resulting from mutations in primary cilium-associated proteins is likely due to cell type specific disturbances in a range of different signalling systems.

We carried out an ENU mutagenesis screen to identify genes with critical functions during early embryonic development [17] and have identified a mutant with a strong primary cilia phenotype. Linkage and candidate gene sequencing identified a missense mutation in the core IFT-A gene *Ift140*. This mutation results in mid-gestation lethality, exencephaly, craniofacial dysmorphism, skeletal malformation and mixed poly- and oligodactyly. A number of these defects can be attributed to disregulated Hedgehog signalling while other defects indicate involvement of additional signalling systems. Based on the identification of an *Ift140* mutation in this mouse model we also sequenced the human orthologue in a cohort of SRP and Jeune syndrome patients. We present the identification of a novel *IFT140* mutation in a case of Jeune syndrome. IFT140 has recently been implicated in Mainzer-Saldino and Jeune syndromes and conditional deletion in mice causes a polycystic kidney phenotype [18], [19], [20]. Together these data confirm the validity of our mouse model of Jeune syndrome and will underpin further investigations into the mechanisms responsible for this condition.

## Results

### *Cauli* is a novel ciliopathy mouse model with a recessive missense mutation in the *Ift140* gene

**Figure 1:**
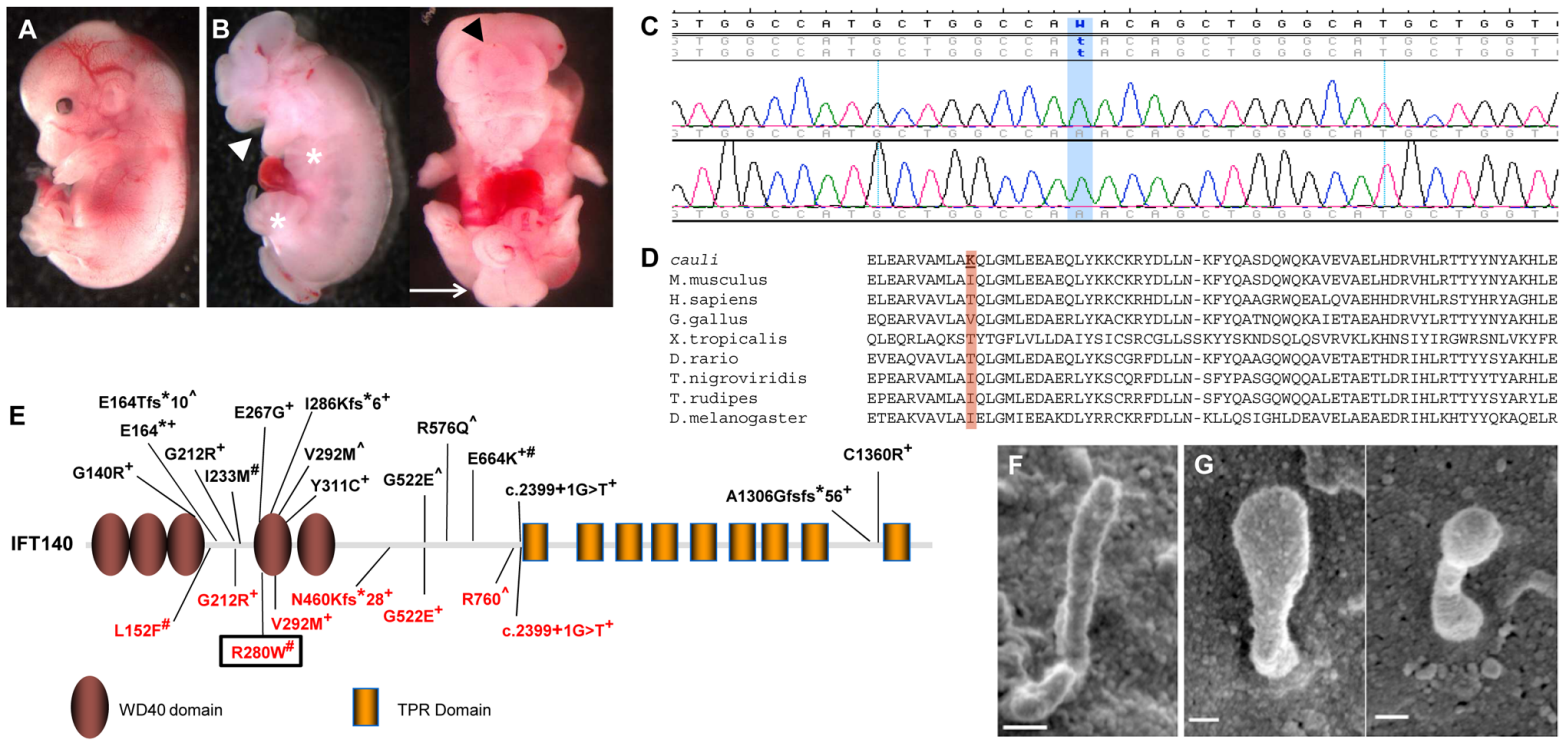
An *Ift140* mutation is responsible for the ciliopathic phenotype observed in *cauli*. Representative E13.5 wildtype (A) and mutant (B) embryos showing exencephaly (black arrowhead), open mouth (white arrowhead), polydactyly (asterisks) and caudal neural tube closure defects (arrow) in mutants. Chromatogram of *cauli* mutant showing the homozygous missense mutation (c.2564T>A) in the Intraflagellar Transport Protein 140 (*Ift140*) gene (C). IFT140 protein alignment showing the isoleucine to lysine substitution at position 855 of the protein in *cauli* and the corresponding amino acid across several species (D). Schematic of the IFT140 protein detailing protein domains, location of *Ift140^cauli/cauli^* mutation and reported human mutations (E). Mainzer-Saldino (black), Jeune asphyxiating thoracic dystrophy (red), ^+^compound heterozygous, ^#^homozygous ^∧^no second mutation identified. Black box represents mutation reported in this paper. Primary cilia from E10.5 *Ift140^+/+^* (F) and *Ift140^cauli/cauli^* (G) limb buds show a severely altered cilia morphology in the mutant.

The *cauli* strain was identified via a comprehensive phenotype-driven ENU screen undertaken to identify novel genes involved in embryogenesis [17]. The vast majority of *cauli* homozygous embryos survive to approximately embryonic day (E) 13.5, however a small proportion has lived beyond this age (n = 10/130). No live embryos were obtained >E16.5. Fully penetrant phenotypes identified in *cauli* mutants include exencephaly (n = 130/130), anopthalmia (n = 130/130) and polydactyly of the hindlimbs (Figure 1B; n = 32/32 embryos analysed at >E12.5). *Cauli* mutants were developmentally delayed compared to wildtype littermates and additional phenotypes observed in these mice include oligodactyly of the forelimbs, gaping mouth, omphaloceole, oedema, curly tail and caudal neural tube closure defects (Figures 1A and B).

Linkage analysis in *cauli* identified a 7 Mb locus on chromosome 17 between markers rs3667809 and rs3684506 harbouring 218 genes. The *Ift140* gene was prioritised as a strong putative candidate due to the similarity of the *cauli* phenotype with other ciliopathy mouse models [21], [22], [23]. Direct sequencing of the *Ift140* gene (NCBI RefSeq transcript NM_134126.3) identified a T to A substitution in exon 19 at position 2564 (Figure 1C; c.2564T>A), resulting in an isoleucine to lysine amino acid change at position 855 of the protein (p.I855K).

We utilized two algorithms designed to assess the potential effect of the isoleucine to lysine mutation on Ift140 function. The prediction output from PolyPhen-2 for the *cauli* Ift140 p.I855K mutation indicates a ‘possibly damaging’ effect of the mutation on protein function, with a PSIC score of 0.503. SIFT prediction output denotes that the mutation is ‘not tolerated’, with a probability of 0.00, projected to be deleterious. Alternative amino acids found at this residue in other species (Threonine and Valine) were all predicted to be ‘tolerated’ by this analysis (Figure 1D). This residue resides within a coiled-coil domain immediately upstream of the first tetratricopeptide repeat (TPR) domain of mouse IFT140, as predicted by the algorithms TPRpred and coils/pcoils [24] (http://toolkit.tuebingen.mpg.de/). Interestingly, secondary structure prediction using the PSIPRED algorithm [25] indicates that the I855K mutation will not disrupt the α-helix in which it is embedded and therefore likely has a subtle impact on the overall structure of Ift140. Thus, the implications of the I855K change for mutant Ift140 function are unclear.

Ultrastructural examination of the primary cilia of E10.5 *Ift140^+/+^* and *Ift140^cauli/cauli^* limb buds by SEM (Figures 1F and G) revealed, that when present, cilia morphology was severely disrupted in *Ift140^cauli/cauli^* mutant embryos. Cilia in these mice were much broader and had a bulbous appearance, consistent with the accumulation of cargo at the tip due to a retrograde transport defect.

### *Ift140^cauli/cauli^* embryos exhibit multiple developmental defects

**Figure 2:**
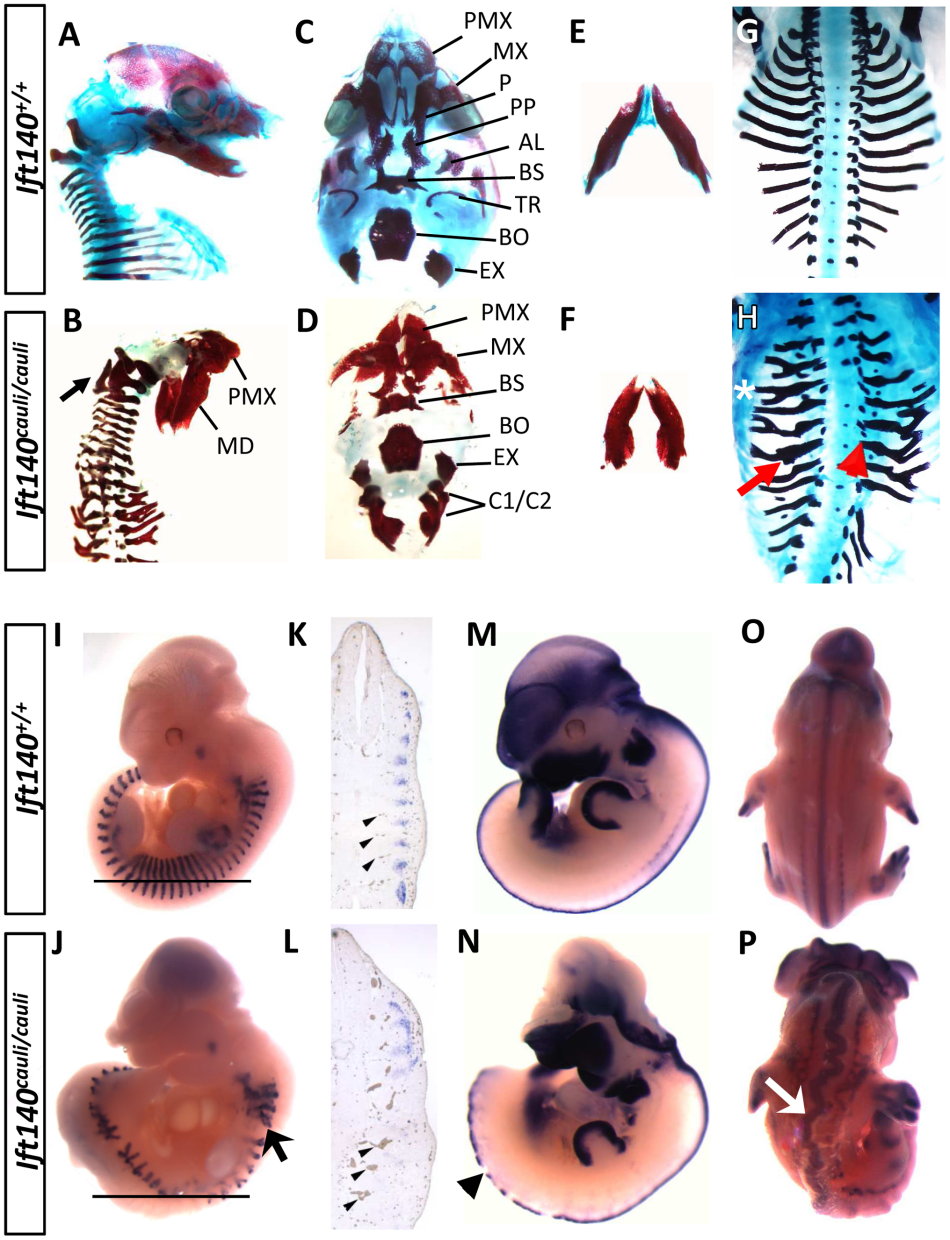
*Ift140^cauli/cauli^* embryos exhibit skeletal, somite and neural tube defects. Morphological and expression analysis of *Ift140^+/+^* and *Ift140^cauli/cauli^* embryos. Lateral view of E16.5 skull in *Ift140^+/+^* (A) and *Ift140^cauli/cauli^* (B) embryos. *Ift140^cauli/cauli^* embryos exhibit fusion of the exoccipital bone and C1/C2 vertebrae (arrow in B). Ventral view of skull base in *Ift140^+/+^* (C) and *Ift140^cauli/cauli^* (D) embryos. *Ift140^+/+^* (E) and *Ift140^cauli/cauli^* (F) mandibles. The normal organisation of the ribs seen in E16.5 *Ift140^+/+^* embryos (G) is severely disrupted in *Ift140^cauli/cauli^* (H) with lateral branching (asterisk), thickened ossified nodules (red arrow) and abnormal costovertebral articulations (red arrowhead). (I–P) *In situ* hybridisation of gene expression patterns of *myogenin* (I–L), *Msx1* (M,N) and *Sox9* (O,P). *Myogenin* staining at E11.5 reveals the highly ordered segmental structure of a *Ift140^+/+^* embryo (I) while in the *Ift140^cauli/cauli^* embryo (J) *myogenin* staining reveals the presence of disorganised and branched somite-derived structures (myotome; arrow). (K,L) Sections of wholemount embryos at the level indicated by the horizontal line in I and J, illustrating the loss of segmental *myogenin* staining and the accumulation of blood within distorted and irregular intersomitic vessels (arrowheads) in *Ift140^cauli/cauli^* embryos. (M,N) *Msx1* expression delineates the dorsal margin of the neural tube in an E11.5 *Ift140^+/+^* embryo (M) but highlights the disrupted neural tube structure in an *Ift140^cauli/cauli^* embryo (arrowhead in N). In addition, the neural tube is convoluted and irregular in appearance, as shown in E12.5 embryos stained for *Sox9* (arrow in P). PMX, premaxilla; MD, mandible; MX, maxilla; P, palatine; PP, palatal process; AL, alisphenoid; BS, basisphenoid; TR, tympanic ring; BO, basioccipital; EX, exoccipital; C1/C2, fused 1^st^ and 2^nd^ cervical vertebrae.

Morphological differences between *Ift140^+/+^* and *Ift140^cauli/cauli^* embryos were analysed using a number of techniques. Skeletal preparations of surviving E16.5 mutant embryos (n = 2) revealed severe craniofacial defects including failure of calvarial development, cervical vertebral fusion and agenesis or hypoplasia of many facial bones (Figures 2A–F). The exoccipital was fused to the first cervical vertebra. The shape of the exoccipital, bassioccipital and basisphenoid, appeared relatively normal. In contrast, the tympanic ring, alisphenoid and palatine were absent or rudimentary while the maxilla and premaxilla were hypoplastic and malpositioned due to the absence of components with which they would normally articulate. Examination of the thoracic skeleton revealed severe rib defects in *Ift140^cauli/cauli^* embryos when compared to the ordered structure seen in *Ift140^+/+^* controls (Figures 2G and H). Although the normal complement of 13 ribs was present, anomalies of the ribs in *Ift140^cauli/cauli^* embryos include abnormal costovertebral articulations (the joints between the heads of each rib and the thoracic vertebrae), lateral bifurcation (branching) and thickened ossified protuberances (exostoses, Figure 2H). Due to the gestational lethality observed in *Ift140^cauli/cauli^* embryos further skeletal analysis was not possible. To further examine the origins of axial defects observed in skeletal preparations, somite patterning was analysed in E11.5 embryos by *in situ* hybridisation with the somatic marker *myogenin* (Figures 2I–L). Consistent with the rib phenotype identified in older embryos, somites in E11.5 *Ift140^cauli/cauli^* mutants were extremely disorganised with a bifid appearance, more evident in somites located in the rostral region (Figures 2J and L). This pattern is in stark contrast to the highly ordered somite pattern seen in *Ift140^+/+^* control embryos (Figures 2I and K). Sections of wholemount embryos confirmed the disruption of normal *myogenin* staining and revealed the accumulation of blood within distended and irregularly located inter-somitic vessels (Figures 2K and L). In addition to the obvious cranial neural tube defects, irregularities in the integrity of the axial neural tube were also revealed by *in situ* hybridisation analysis. *Msx1* marks the dorsal-most neural tube cell population and in E11.5 wildtype controls appears as a continuous line of staining, while in *Ift140^cauli/cauli^* mutants staining is irregular and discontinuous (Figures 2M and N). Analysis of older embryos (E12.5) with a *Sox9 in situ* probe reveals the straight, aligned neural tube of *Ift140^+/+^* embryos (Figure 2O) in comparison to the irregular and extremely convoluted neural tube seen in *Ift140^cauli/cauli^* mutants (Figure 2P).

**Figure 3:**
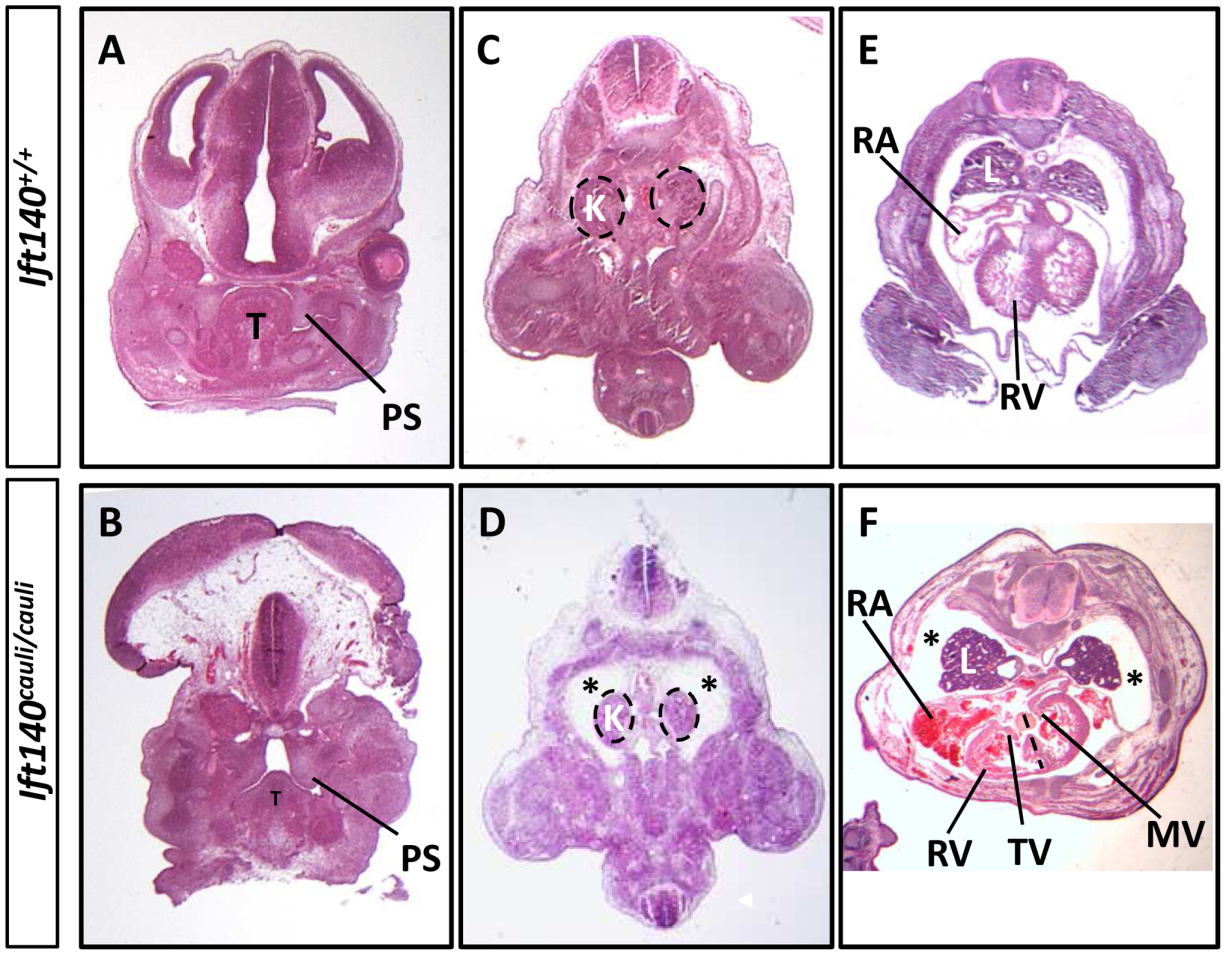
*Ift140^cauli/caul^* mutants show palate defects, hydrops fetalis and malformation of the lungs and heart. Coronal sections of E13.5 *Ift140^+/+^* (A) and *Ift140^cauli/cauli^* (B) palates, highlighting hypoplastic palatal shelves in *Ift140^cauli/cauli^* embryos. Transverse sections of E13.5 *Ift140^+/+^* (C and E) and *Ift140^cauli/cauli^* (D and F) embryos at the level of the kidneys (C and D) and heart (E and F). *Ift140^cauli/cauli^* mutant embryos have grossly normal kidneys but show accumulation of fluid around the kidneys and lungs (asterisks in D and F), which is not evident in *Ift140^+/+^* controls (C and E). The lungs of *Ift140^cauli/cauli^* embryos are also abnormal in shape (F), unlike the cone-shaped lobes seen in *Ift140^+/+^* controls (E). The atrioventricular valves of the heart are well formed in *Ift140^+/+^* embryos (E), but both the tricuspid and mitral valves are abnormal in *Ift140^cauli/cauli^* mutants (F). *Ift140^cauli/cauli^* embryos appear to have ventricular hypotrophy and the interventricular septum is not well formed (depicted by dashed line in F). An irregular accumulation of blood can also be seen in the atria and ventricles of *Ift140^cauli/cauli^* mutants (F). T, tongue; PS, palatal shelf; K, kidneys; L, lung; RA, right atrium; RV, right ventricle; TV, tricuspid valve; MV, mitral valve.

*Ift140^cauli/cauli^* mutant embryos begin to die at E13.5 during the stage when palatal fusion occurs. It is therefore difficult to determine if older mutants have a specific palate fusion defect or whether palate fusion is impeded due to a global disruption of growth preceding death. To investigate the impact of the *cauli* mutation on palatal development we performed histological analysis at E13.5 in healthy individuals. Coronal sections through the palatal shelves of *Ift140^+/+^* and *Ift140^cauli/cauli^* embryos at E13.5 identified hypoplastic palatal shelves in *Ift140^cauli/cauli^* mutants, suggesting that palate fusion would be impeded in older mice (Figures 3A and B). Histological analysis of E13.5 *Ift140^+/+^* and *Ift140^cauli/cauli^* embryos also highlighted numerous deformities of the internal organs (Figures 3C–F). Although the kidneys in *Ift140^cauli/cauli^* embryos appear grossly normal and comparable to *Ift140^+/+^* kidneys, an unusual cavity is evident around these structures, consistent with an accumulation of fluid (Figures 3C and D). The lungs are severely misshapen in *Ift140^cauli/cauli^* embryos with a rounded appearance, unlike the cone-shape lobes clearly evident in *Ift140^+/+^* sections (Figures 3E and F). Analysis of heart morphology indicates an acute heart phenotype in *Ift140^cauli/cauli^* embryos when compared to the normal heart structure of *Ift140^+/+^* controls (Figures 3E and F). The unusual accumulation of blood within the atria may be a consequence of the malformed tricuspid and mitral atrioventricular valves and the irregular interventricular septum formation identified in *Ift140^cauli/cauli^* embryos (Figure 3F).

### Molecular signalling is disrupted in *Ift140^cauli/cauli^* limb buds

**Figure 4:**
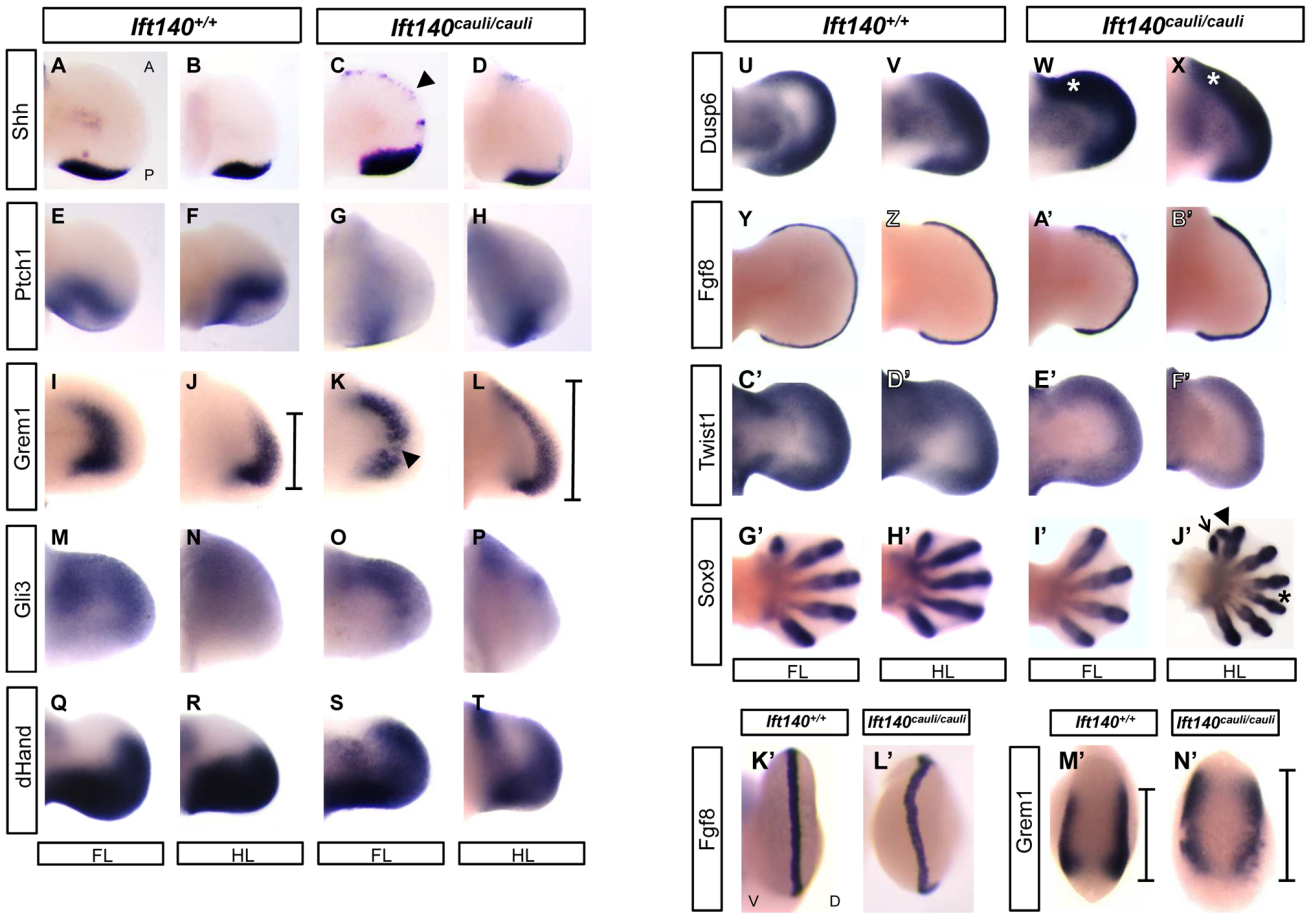
Molecular signalling is disturbed in *Ift140^cauli/cauli^* embryos. WISH analysis of the forelimbs and hindlimbs of *Ift140^+/+^* and *Ift140^cauli/cauli^* embryos. Dorsal view of fore- and hindlimb buds (A–J′), where anterior is always to the top of the image. Distal view of forelimb buds (K′–M′), where dorsal side is facing to the right of the image. Arrowhead in (C) indicates ectopic *Shh* expression domain. Bars in (J,L and M′,N′) indicate anterior-posterior extent of *Grem1* expression. Arrowhead in (K) indicates disruption in *Grem1* expression in mutant forelimb. Asterisk in (W and X) indicates elevated anterior *Dusp6* expression. Arrow, arrowhead and asterisk in J′ indicate a single ectopic digit, bifid ectopic digit and proximal syndactyly respectively. A, anterior; P, posterior; D, dorsal; V, ventral; FL, forelimb; HL, hindlimb. All embryos are E11.5 except G′–J′ which are E13.5.

The Shh/Grem1/Fgf loop is a key signalling system responsible for controlling the outgrowth and patterning of the limb [26]. Components of this signalling system were analysed in *Ift140^cauli/cauli^* limb buds by *in situ* hybridisation (Figure 4). Many members of the Shh/Grem1/Fgf signalling loop show disrupted expression between *Ift140^+/+^* control and *Ift140^cauli/cauli^* mutant limb buds at E11.5–13.5 days of development (Figure 4). Ectopic *Shh* expression is evident along the anterior margin of *Ift140^cauli/cauli^* limb buds (Figures 4A–D). Expression of *Ptch1*, the *Shh* receptor and a *Shh*-responsive gene, is reduced posteriorly but expanded anteriorly across limb buds of *Ift140^cauli/cauli^* mutants consistent with the presence of anterior Shh (Figures 4E–H). The distinct expression pattern of the Bmp antagonist *Grem1* seen in *Ift140^+/+^* limb buds is altered in *Ift140^cauli/cauli^* mutants (Figures 4I–L, M′ and N′). *Grem1* expression appears weaker in mutants compared to controls but is expanded both anteriorly and posteriorly. *Gli3*, a major mediator of *Shh* signalling in the limb, is spatially restricted in the anterior portion of the fore- and hindlimb buds of *Ift140^cauli/cauli^* embryos (Figures 4M–P). Expression of *dHand*, which has a major role in limb patterning at least in part through a reciprocal regulatory interaction with *Shh*, is reduced posteriorly but expanded into the anterior limb bud (Figures 4Q–T). *Dusp6* (*Mkp3*) is a downstream mediator of Fgf signalling. Expression of *Dusp6* is elevated across the anterioposterior axis but is anteriorly expanded (Figures 4U–X). Consistent with this, while *Fgf8* expression is maintained in a distinct pattern outlining the AER at the distal tip of wildtype and *Ift140^cauli/cauli^* limb buds, expression is elevated in mutants (Figures 4Y–B′). In addition, *Fgf8* staining also indicates that in *Ift140^cauli/cauli^* mutants there is a disruption in the location of the AER at the border between the dorsal and ventral limb bud ectoderm relative to *Ift140^+/+^* controls (Figures 4K′ and L′). *Twist1* has been demonstrated to have a central role in patterning the limbs and in particular has been shown to regulate *Shh* expression. Given the alteration in *Shh* expression in *Ift140^cauli/cauli^* mutants it was important to examine the level and location of *Twist1* expression. Interestingly, expression of *Twist1* in *Ift140^cauli/cauli^* mutants did not appear to undergo any substantial spatial alteration but did appear to be repressed in *Ift140^cauli/cauli^* embryos relative to controls (Figures 4C′–F′). Analysis of digit number was often difficult in younger embryos when overt signs of digit outgrowth where not yet apparent. *Sox9* expression highlights the presence of digital rays within the autopod revealing oligodactyly, proximal and distal branching of digits and polydactyly in *Ift140^cauli/cauli^* embryo limb buds (Figures 4G′–J′).

### Abnormal epithelial morphology in *Ift140^cauli/cauli^* limb bud ectoderm

**Figure 5:**
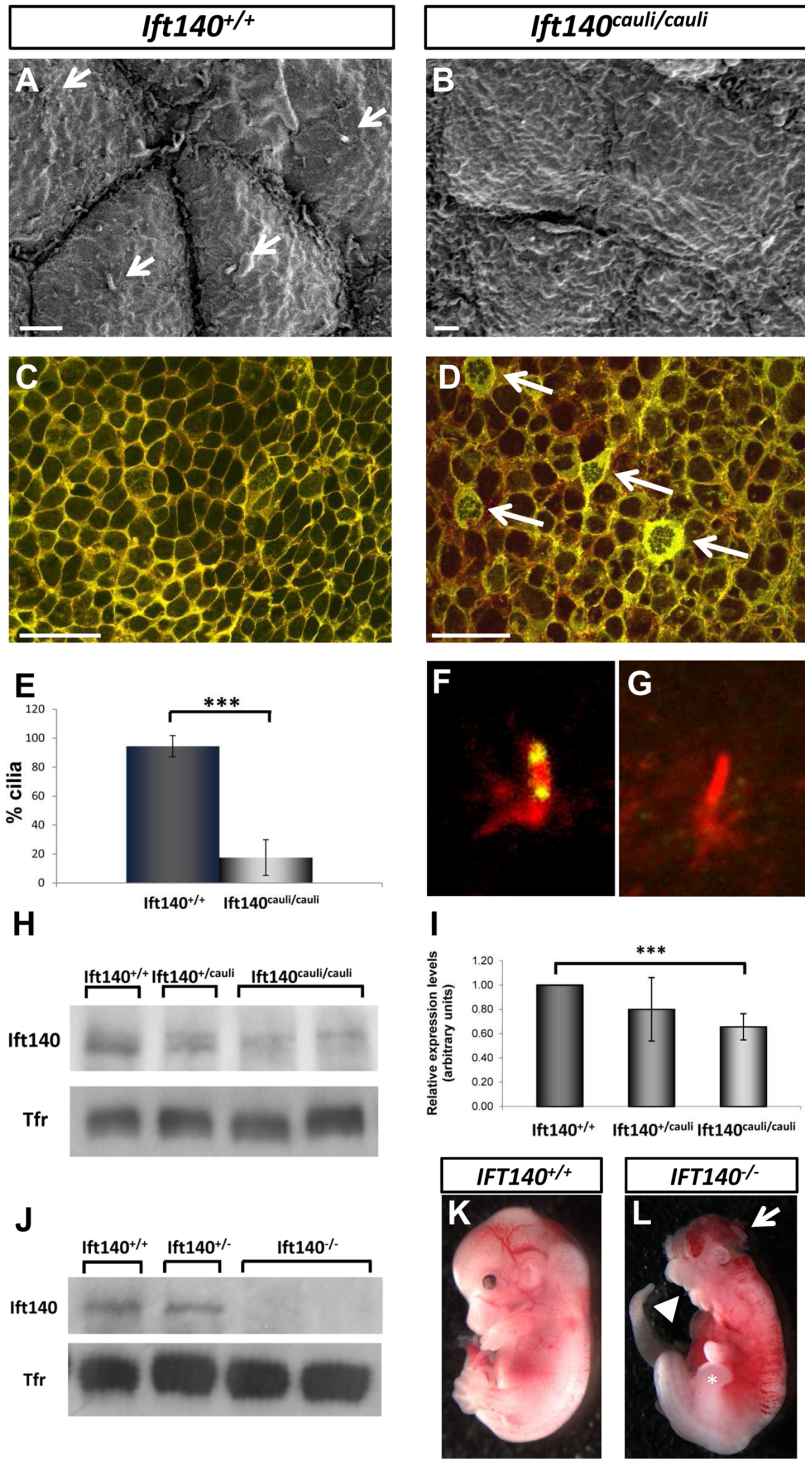
Epithelial cellular architecture and levels of Ift140 are altered in *Ift140^cauli/caul^* mutants. Scanning electron micrographs and immunohistochemistry of epithelia from E10.5 *Ift140^+/+^* (A and C) and *Ift140^cauli/cauli^* (B and D) limb buds. The rigid cellular architecture seen in wildtype limbs is highly disrupted in the mutant, as evidenced by the lack of cilia (compare arrows in A), the presence of thick, disorganised cell junctions (arrows in D) and the more diffuse E-cadherin staining in the mutant (D). Overlay of E-cad (green) and phalloidin (red) in *Ift140^+/+^* (C) and *Ift140^cauli/cauli^* (D) epithelium. Cilia counts identify a significant decrease in cilia (***p = 2.05×10^−7^) in limb buds of *Ift140^cauli/cauli^* when compared to *Ift140^+/+^* controls (E). Ift140 can be detected at the base and tip of wildtype limb bud cilia (F) but it undetectable in the majority of mutant cilia (G). Western blot analysis shows a reduction of Ift140 protein levels in *Ift140^cauli/cauli^* tissue when compared to control and heterozygous samples (H), and a significant reduction of *Ift140* transcript levels (***p = 0.0026) in *Ift140^cauli/cauli^* mutant embryos (I). Embryos harbouring a homozygous *Ift140* null allele show a complete lack of Ift140 protein by western blot (J) and exhibit identical phenotypes to those identified in *Ift140^cauli/cauli^* embryos (K,L), including exencephaly (white arrow), open mouth (white arrowhead) and an expanded hindlimb field (asterisk). Scale bar; 2 µM (A and B), 30 µM (C and D).

Scanning electron microscopy of E10.5 *Ift140^+/+^* control and *Ift140^cauli/cauli^* mutant limb buds identified a defect in the epithelial cellular architecture (Figures 5A and B). In *Ift140^+/+^* control limb buds, epidermal ectodermal cells have distinct, clearly defined cellular borders (Figure 5A). In contrast, individual cells in *Ift140^cauli/cauli^* mutants have poorly defined cellular borders and individual cells are difficult to identify from surface topology (Figure 5B). In addition, while the primary cilia are easily identified in >90% of wildtype cells, <20% of *Ift140^cauli/cauli^* mutant epithelial cells harbour an identifiable cilium in SEM images (Figures 5A, B, E).

Epithelial cadherin (E-cad) is a component of cell to cell adherens junctions and is required for the maintenance of tight intracellular junctions and oriented alignment of the cytoskeletal networks [27]. Cytoskeletal filamentous actin (F-actin) also localises to these adhesion junctions and is important for regulating tight junction functions within and between cells [28]. In wildtype epithelium, E-cad and F-actin closely co-localise at discrete cellular junctions clearly defining cell borders (Figure 5C). However in *Ift140^cauli/cauli^* mutants adherens junctions become diffuse and dispersed in the mutant epithelium and E-cad and F-actin become partially delocalised (Figure 5D). In a proportion of *Ift140^cauli/cauli^* epithelial cells, very high levels of E-cad can be seen in a thick band around the cell periphery and a network of E-cad and F-actin appears within the cytoplasm of many of these cells (Figure 5D).

The prediction that the I855K mutation is likely to have a modest impact on structure of Ift140 prompted us to investigate the impact of the *cauli* missense mutation on Ift140 function by examining the distribution of Ift140 in the primary cilia of limb bud epithelial cells. In wildtype epithelium Ift140 localises to the base and tip of the primary cilium (Figure 5F). In *Ift140^cauli/cauli^* mutant epithelium there were undetectable levels of Ift140 in the majority of cilia (Figure 5G) while in rare cases (∼5%) a small amount of Ift140 could be seen in association with a mutant cilium. The lack of detectable Ift140 in mutant cilia prompted an investigation of the levels of Ift140 expressed by mutant cells. Western blot of protein extracted from whole E11.5 embryos indicated a reduction of Ift140 protein to approximately 85% of wildtype levels in *Ift140^cauli/+^*embryos and approximately 30% of wildtype levels in *Ift140^cauli/cauli^* embryos (Figure 5H). We then examined the level of *Ift140* mRNA in *Ift140^cauli/cauli^* mutant and wildtype littermates by real-time PCR and observed an approximately 30% decrease in *Ift140^caul/caulii^* embryos compared to wildtype controls (Figure 5I). This data suggests that the *Ift140^cauli/cauli^* phenotype results from haploinsufficiency. To examine this hypothesis further, we generated *Ift140* null embryos. Western blot analysis confirmed the loss of Ift140 in this model (Figure 5J) and analysis of *Ift140^−/−^* embryos demonstrated an identical phenotype to that observed in *Ift140^cauli/cauli^* embryos (Figures 5K and L) with the exception that *Ift140^−/−^* embryos die at around E11.5, two days earlier than *Ift140^cauli/cauli^* embryos. The similarity of the phenotypes in the two models supports the theory that the cauli phenotype results from haploinsufficiency.

### An *IFT140* mutation identified in a human patient with Jeune Syndrome

**Figure 6:**
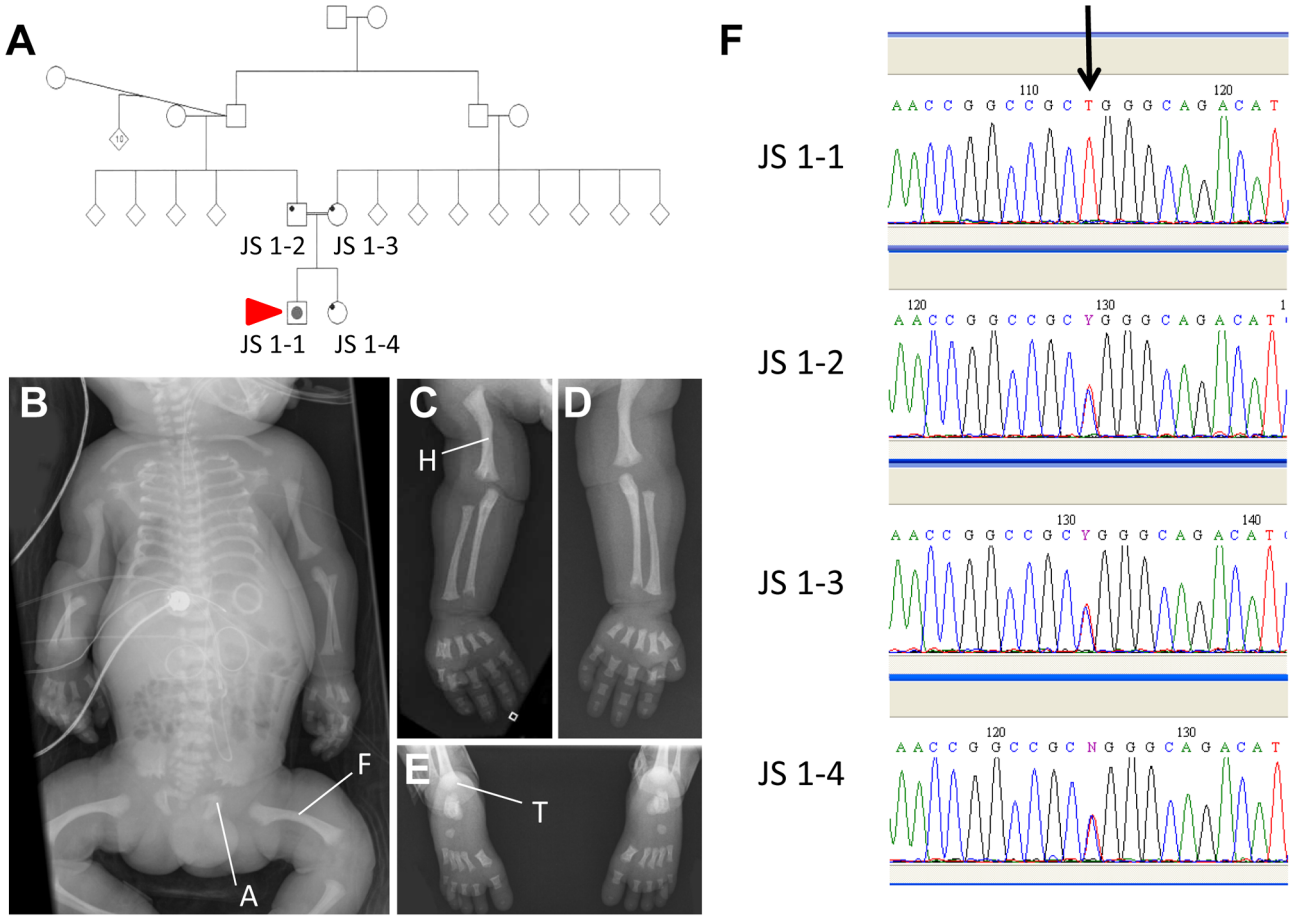
An *IFT140* mutation identified in a Jeune Syndrome patient. Family pedigree showing the relationship between JS1-1, JS 1-2, JS 1-3 and JS 1-4 (A; red arrow indicates the proband). Whole body (B), right (C) and left (D) upper limb and feet (E) radiographs from JS1-1. Note the short ribs, short long bones with bowed humeri and femora, “trident” appearance of the acetabular roofs and metaphyseal irregularity (B), short long bones with metaphyseal cupping and bowed humerus (C and D), and metaphyseal cupping and advanced tarsal bone ossification for age (E). A, acetabulum; F, femur; H, humerus; T, tarsus. Sequence chromatogram identifying the C>T homozygous mutation in JS1-1 (F; arrow), and heterozygous mutation in immediate family members.

Patient JS 1-1 was the product of the first pregnancy to healthy, first-cousin Sudanese parents (Figure 6A). There was no extended family history of skeletal disorders. Short limbs (less than 5^th^ centile for gestational age) and a short chest cage were noted on a third trimester ultrasound performed for growth assessment. No other abnormalities were observed at this time. The couple were counselled about a likely poor prognosis given the degree of thoracic hypoplasia. The child was delivered at 37 weeks of gestation with a birth head circumference of 37 cm (>98^th^ centile) and birth length of 48 cm (50^th^ centile). The baby was in poor condition and required high frequency ventilation with high peak pressures (32 mm Mercury) due to pulmonary hypoplasia and hypertension. On examination, the child had short square fingers and hands with five digits each, low nasal bridge, normal ears and palate, and hepatomegaly. A small muscular ventricular septal defect was found on echocardiography, and ultrasound of the enlarged liver showed normal hepatic texture. Normal liver and renal function was documented. The baby deteriorated, despite ventilation, and died aged 7 days. Post mortem radiography was obtained and revealed short long bones, with metaphyseal cupping, bowed femora and humeri, short ribs and thorax, trident appearance to the acetabular roofs, and advanced tarsal bone ossification (Figures 6B–E). These features are considered typical of classic Jeune asphyxiating thoracic dystrophy) syndrome.

Sequencing the *IFT140* gene in JS 1-1 identified a novel C>T homozygous mutation at position 838 in this gene that was heterozygous in both parents and the unaffected sibling (Figure 6F), and was not present in a cohort of 90 healthy controls. The Arg to Trp amino acid change at position 280 resulting from this variation attained a PSIC score of 0.972, indicative of a ‘probably damaging’ effect of the mutation on protein function.

## Discussion

There are now at least 12 clinical entities defined as ciliopathies [13]. Given the ubiquity of the primary cilia and the number of signalling systems that make use of the cilium as a site in which to deploy receptors and other signalling components, the number of congenital and acquired conditions attributed to defects in cilia function is expected to rise. Mutations in *IFT140* have recently been identified in the skeletal ciliopathies Mainzer-Saldino (OMIM 266920) and Jeune (OMIM 208500) syndromes [this paper and 18], [19]. These mutations do not appear to cluster to a particular domain within the IFT140 protein (Figure 1E). While there is significant phenotypic overlap between these two syndromes, including radiological and renal findings, there are also significant differences, such as the existence of skin pigmentation anomalies in Mainzer-Saldino syndrome and polydactyly in Jeune syndrome. These phenotypic differences may be attributable to the impact of specific mutations on the function of IFT140 within the cilium. Mutation of *IFT80*, a component of the anterograde transport complex B, has also been demonstrated to cause Jeune syndrome [29]. IFT140 resides within the retrograde complex A, thereby suggesting substantially different consequences for the mechanisms of transport within the cilia. Further, mutations in additional IFT components including TTC21B/IFT139, DYNC2H1 and WDR19/IFT144 have been reported in association with Jeune syndrome [30], [31], [32], [33]. Similarly, IFT-A complex mutations have been associated with Sensenbrenner syndrome, a condition with substantial clinical overlap with Jeune syndrome [32]. This suggests that the common phenotypes resulting from each of these mutations are a result of defective cilia function rather than the specific mutation and indicates that this sub-group of skeletal ciliopathies may represent a spectrum of malformations rather than distinct syndromes. There are clear limitations to this view of skeletal ciliopathies since IFT-A complex mutations also lead to clinically distinct entities such as isolated nephronophthisis [32]. These complexities highlight the need to understand the developmental mechanisms underlying specific skeletal ciliopathies through study of appropriate animal models.

The *Ift140^cauli/cauli^* mutant mouse phenocopies many of the features of Jeune syndrome although the mouse phenotype is generally more extreme than in humans. The severe dysmorphology seen in *Ift140^cauli/cauli^* embryos is incompatible with continued development beyond mid-gestation in the majority of cases. Despite considerable efforts only a very small number of embryos have been obtained beyond E15.5 and in these cases severe skeletal malformation was observed. Skeletal phenotypes include severely disorganised ribs with extensive exostoses, vertebral and palatal defects and agenesis/hypoplasia of the craniofacial skeleton. These phenotypes are broadly consistent with those observed in Jeune syndrome cases including the case presented here. The range of phenotypes observed in *Ift140^cauli/cauli^* embryos is also broadly similar to that exhibited by other IFT-A mutant mice [23], [34], [35] consistent with the concept that defective cilia function underpins the dysmorphology observed in each case.

The severity of the *Ift140^cauli/cauli^* mutant phenotype may also reflect the nature of the Ift140 mutation in this model. While the *cauli* strain arose following a missense mutation in *Ift140* we have observed a substantial reduction in both protein and RNA levels in mutant tissues. These data together with the identical phenotype observed in *Ift140^cauli/cauli^* and *Ift140^−/−^* embryos suggests that the *cauli* phenotype results from loss of Ift140 rather than altered function following amino acid substitution. Ift140 is an essential component of the IFT-A complex and retrograde transport will be severely compromised in its absence. Thus, it is likely that much of the developmentally important signalling normally mediated through the primary cilia is compromised in *Ift140^cauli/cauli^* embryos resulting in the devastating phenotype observed.

A missense mutation would normally not be expected to result in RNA degradation and it is not clear why the *Ift140^cauli/cauli^* mutation results in loss of one third of the *Ift140* RNA population. One possible explanation for this observation is that the c2564T>A mutation, which is 17 bp upstream of the 3′ splice site in exon 19, leads to an abnormal splicing event. It is likely that this would introduce a nonsense mutation downstream of the splicing event and the aberrantly spliced RNA would then be subject to nonsense mediated decay [36]. In support of this, the *cauli* mutation disrupts predicted overlapping SRSF5 and SRSF6 binding sites raising the possibility that an exonic splice enhancer (ESE) could be impacted [37], [38]. ESEs have been shown to be an integral part of both ubiquitous and alternate splicing mechanisms and exonic mutations disrupting them can result in exon-skipping and the generation of non-sense mutations [39], [40]. There are a growing number of conditions attributable to mutation of an ESE [41], [42], [43], [44], [45], [46].

The somites in *Ift140^cauli/cauli^* mutant embryos exhibit a dramatic loss of normal patterning, exhibiting both branching and irregular distribution. There does not appear to be an obvious loss of individual somites suggesting that segmentation of the lateral plate mesoderm proceeds relatively normally. However, irregular morphology of somites suggests that some aspects of somitogenesis, such as the mesenchymal to epithelial transition, may not be regulated appropriately. These somite patterning anomalies are likely responsible for the later rib defects in *Ift140^cauli/cauli^* since the ribs are derived from the dermomyotome, a dorsolateral compartment of the somites [47]. In addition, the existence of bony protuberances emanating from many *Ift140^cauli/cauli^* mutant ribs suggests a disregulation of osteogenesis. *Ift140^cauli/cauli^* mutants exhibit elevated *Ptch1* expression within the flank indicating that rib exostoses may result from altered Hh signalling. Mutants are significantly growth retarded relative to littermates, which is most likely related to the mid-gestational demise of most mutants but may also be affected by reduced axial growth. *Ift140^cauli/cauli^* mutants exhibit a convoluted neural tube, a phenotype thought to be caused by disruption of coordination between neural tube and axial elongation. Convergent extension defects have been linked to the disruption of primary cilia formation previously [48], [49]. Disrupted convergent extension during neurulation results in failure to narrow the mid-line thereby promoting neural tube closure defects [50]. *Ift140^cauli/cauli^* mutants exhibit both cranial and lumbar level neural tube closure defects and it will be interesting in future studies to examine neurulating *Ift140^cauli/cauli^* embryos for defects in convergent extension activities.

The polydactylous phenotype observed in *Ift140^cauli/cauli^* mutants is similar to that reported in other cilia mutant mouse lines and is highly reminiscent of patterning defects exhibited by a number of Shh signalling mutants [21], [34], [51]. The primary cilia function downstream of Shh and IFT proteins have been demonstrated to be essential for all Shh signalling downstream of Smo [52]. The relative distribution of Gli3 activator and Gli3 repressor across the anterior-posterior axis of the limb bud is a crucial determinant of autopod patterning [53]. Shh signalling inhibits the proteolytic cleavage of full length Gli3 into the shorter Gli3 repressor form which is both dependent on IFT and occurs within the primary cilia [54], [55], [56]. Loss of IFT results in reduced production of Gli3 repressor and consequently an increase in activation of genes such as *Grem* and *dHand*, whose expression is linked to Shh and Gli3 action. Our analysis demonstrates that expression of these genes is expanded into the anterior of the autopod in *cauli* embryos where Gli3 repressor normally predominates, indicating that a loss of Gli3 repression has occurred. The ectopic expression of *Ptch1* in the anterior limb bud is consistent with previously described IFT-A mutants [23], and may result from Gli activator effects due to reduced Gli cleavage. An altered ratio of Gli3 repressor to the full-length activator form across the limb paddle results in production of a variable number of additional digits in a number of different mouse models [51], [57], [58] and very likely underlies the polydactyly phenotype in cauli embryos.

The expression of *Shh* is regulated by a complex network of transcription factors. Ectopic expression of *dHand* similar to that observed in the anterior limb bud of *cauli* embryos has been shown to be sufficient to induce ectopic *Shh* [59]. In addition, Twist1 has been shown to repress *Shh* expression in the anterior limb bud and *Twist1* null mice have polydactyly [60]. We observed a repression of *Twist1* in *Ift140^cauli/cauli^* mutants and it is therefore possible that the reduction in *Twist1* levels is responsible for releasing the repression of Shh in the anterior limb bud. Further experimentation will be required to establish if this is the case and what role these changes have on autopod morphology.

Polydactyly is observed in 100% of *Ift140^cauli/cauli^* hindlimbs and 50% of forelimbs. On the other hand, approximately one quarter of *Ift140^cauli/cauli^* forelimbs exhibit oligodactyly while it is never seen in the hindlimbs. The variable phenotype between fore- and hindlimbs is difficult to reconcile and suggests that inherent differences in signalling or temporal differences in developmental mechanisms between fore- and hindlimbs result in disparate outcomes. While the co-existence of both oligodactyly and polydactyly within forelimb autopod appears contradictory, especially in light of the highly penetrant polydactyly in the hindlimb autopod, it is important to note that digit number is a continuum and that the final outcome is the result of a complex interplay of transcriptional influences. Mixed forelimb oligo/polydactlyly is also observed in mice with reduced *Twist1* levels [61]. *Twist1* is at the heart of a transcriptional network involved in the control of *Fgf* and *Shh* signalling within the developing limb [60]. Partial loss of *Twist1* results in reduced growth of the posterior limb bud and an expansion of the anterior limb bud due to ectopic anterior *Shh* expression [61]. The ectopic anterior Shh also posteriorises anterior digits and the pattering of the resulting autopod is highly variable. It is unclear why there is incomplete penetrance of each of these outcomes but this may result from stochastic variation in the level of *Shh* and *Fgf* signalling. We observe anterior ectopic *Shh* and it is possible that a similar mechanism operates in the *Ift140^cauli/cauli^* forelimb. In particular, given the likelihood that the *cauli Ift140* mutation primarily disrupts IFT rather than any specific impact on Hh signalling, differences in the level of transport during development could provide significantly variable signalling outcomes. Defects in retrograde transport have been demonstrated to result in the accumulation of large amounts of IFT cargo along the length of the axoneme [12] which likely blocks even residual levels of transport. However, the primary cilium is disassembled and reformed anew during each cell cycle and it is possible that a higher level of signalling is achieved in the newly formed cilium and that this declines as the amount of stranded cargo increases. Since rapid cell proliferation is a hallmark of embryonic development, the opportunity for differences in the level of Hh signalling is high, especially with respect to generating differential outcomes between rapidly and slowly proliferating populations.

Motile cilia function at the node, the embryonic organiser, and help determine cardiac left-right asymmetry by the generation of directional flow of signalling components [62]. Looping of the heart tube is the first visible manifestation of the consequences of nodal signalling during breaking bilateral symmetry. IFT is required in both primary and motile cilia and as such it is likely that mutation of *Ift140* disrupts ciliary transport in the motile cilia of the node. The consequences of disrupted IFT for motility are unclear but 50% of mice with immotile nodal cilia are able to generate normal left-right asymmetry. This suggests a mechanism of signalling in nodal cilia that are normally motile that is independent of the directional flow of signalling components in establishing asymmetry [63]. In addition, cilia have been found in developing heart tissue of E9.5–E12.5 mouse embryos [64]. Shh generated by the pulmonary endoderm has been shown to be important in the specification of progenitors required for atrial and pulmonary trunk septation and disruption of this signalling results in both atrial and atrioventricular septation defects [65]. It is therefore likely that mutation of *Ift140* impacts on cardiogenesis both during the looping phase and during later septation events.

The early demise of *Ift140^cauli/cauli^* mutant embryos precludes an analysis of a number of issues that are likely to result from *Ift140* mutation. A targeted deletion of *Ift140* in collecting ducts results in polycystic kidneys beginning around postnatal day 5 [20] consistent with the previous association of *IFT140* mutations with a range of kidney pathologies including cysts [19]. *Ift140^cauli/cauli^* mutant kidneys appear normal at E13.5 which excludes agenesis but is too early to assess more typically IFT-A associated defects. Similarly, the *IFT140* mutation described here in the patient with Jeune syndrome did not result in any obvious kidney dysfunction but this may be due to the early post-natal lethality of the condition in this case. An *Ift144* mutation has been shown to result in craniofacial defects such as cleft palate [23] but *Ift140^cauli/cauli^* mutants die around the stage at which palate fusion occurs. Since *Ift140^cauli/cauli^* mutants are often growth retarded relative to littermates it is likely that palatal shelf elevation and fusion could be delayed making it difficult to determine if *Ift140* mutation results in cleft palate. Similarly, mid-gestational lethality of *Ift140^cauli/cauli^* mutants makes it difficult or impossible to study the impact of *Ift140* mutation on a range of other organ systems. Further analysis of these issues will require the use of conditional *Ift140* alleles such as the one presented here, the value of which has already been demonstrated in the study of kidney pathology [20].

The data presented here describes the first *Ift140* mouse model of Jeune syndrome. We support this with the identification of a recessively inherited *IFT140* mutation in a patient with Jeune syndrome. The difference in severity of the phenotype between mice and humans harbouring IFT140 mutations makes a direct comparison difficult. However, this unique ENU-induced *Ift140^cauli/cauli^* mouse model will continue to provide insights into the mechanisms controlling normal limb and skeletal development and the congenital anomalies that arise when these signalling systems are perturbed. These insights will be invaluable in increasing our understanding of the origins of Jeune syndrome and related skeletal ciliopathies and provide a foundation for further analysis of the signalling systems regulated through the primary cilium.

## Materials and Methods

### Mice

Overt craniofacial and limb phenotypes were identified in *IFT140^cauli/cauli^* embryos obtained from a large-scale ENU mutagenesis program at the Australian Phenomics Facility, Canberra. Embryonic stem cell clones and chimeric mice were generated from *Ift140*-targeted ES cell line EPD0073_5_G01 (www.komp.org) as part of the Australian Phenome Network, “ES Cell to Mouse” initiative. *Ift140^−/−^* embryos were generated by crossing mice heterozygous for the floxed Ift140 allele with a CMV-Cre deletor strain and then back crossing *Ift140^fl/−^* progeny. All mouse procedures were approved by the Murdoch Children's Research Institute Ethics Committee, MCRI AEC #A647, #A684 and #728. All experiments involved harvest of embryos and mice were culled according to MCRI SOP#21. Embryos were then cooled and placed into fixative.

### Mapping and mutation analysis

The *cauli* line was maintained on an inbred C57BL6/J background and outcrossed to the C3H/HeH mapping strain. Linkage analysis was carried out using 13 F2 progeny using a panel of fluorescent microsatellite markers to localise the mutation to a specific chromosomal interval. Using the UCSC genome browser [66] the linkage interval was examined for putative genes and top candidate genes sequenced. DNA from affected *cauli* embryos were screened for mutations in *Ift140* (NM_134126) by sequencing all exons, intron/exon boundaries and most of the 5′ and 3′ untranslated regions.

### PCR, sequencing and genotyping

Gene-specific primers were designed for amplification of all 30 exons of the murine *Ift140* gene and DNA amplified with AmpliTaq (Roche) DNA Polymerase using standard PCR cycling conditions with an annealing temperature of 55°C. PCR products were sequenced with a BigDye v3.1 Terminator Cycle Sequencing Kit (Applied Biosystems) and products read using an ABI 3130xl capillary genetic analyser (Applied Biosystems). Sequencing chromatograms were compared to the published gDNA sequence and any differences identified and determined for potential pathogenicity using PolyPhen-2 and SIFT [67], [68]. Conservation of Ift140 protein sequence was analysed using Clustal W [69].

Adult mouse ear clips/tails and embryonic sacs were digested for genotyping in 0.4 mg/ml Proteinase K in Proteinase K buffer (10 mM Tris-HCl, pH 7.5; 10 mM NaCl; 10 mM EDTA, pH 8; 0.5% SDS) overnight at 55°C. DNA was then purified by isopropanol extraction for amplification and High Resolution Melt (HRM) analysis of *Ift140* exon 19 using primers *Ift140*.genF: 5′-CCAGAACTGGAAGCCAGAGT and *Ift140*.genR: 5′- CTGGGACTACTCACCAGCAT. For HRM, a PCR reaction was performed in 10 µl containing 2.5 µl of 1/500 diluted gDNA, 5 µl Lightcycler480 High Resolution Melting Master Mix (Roche), 3 mM MgCl_2_ and 0.2 µM of each primer. DNA was amplified for 45 cycles of 95°C for 10 sec, 62°C for 15 sec, 72°C for 5 sec. HRM melting curve data were obtained by slowly increasing the temperature from 75°C to 90°C at a rate of 0.02°C per second. Results were analysed by comparing each melt curve to control genotype DNA. Tissue from Ift140 null mice was extracted and amplified using a common forward PCR primer and two different allele specific reverse primers to produce unique products for each genotype. Primers are detailed in Table 1.

**Table 1:** Oligonucleotide primers used in PCR.

Allele/Gene	Template	Forward Primer	Reverse Primer	Size
**Ift140 +/+**	gDNA	CAACTACTAACGAACTGCAACC	CTGTCTTCCCACTCAACTTTACC	**482 bp**
**Ift140 (floxed)**	gDNA		CAACGGGTTCTTCTGTTAGTCC	**241 bp**
**Ift140 (non-deleted)**	gDNA	CGGTCGCTACCATTACCAGT	AGGAGGAGGGAGAGTTTTGG	**657 bp**
**Ift140 (deleted)**	gDNA		GTGTACCTGCCGTCCTGATT	**485 bp**
**TBP**	cDNA	CATCTCAGCAACCCACACAG	GACTGCAGCAAATCGCTTG	**284 bp**
**Ift140**	cDNA	TGGGCCCAGTATCTTGAGAG	CTAAGCCGTTCTCCTTGCAC	**274 bp**

### *In silico* analysis of *cauli Ift140* mutation

Pathogenicity of the *cauli* p.I855K mutation was estimated using PolyPhen-2 (http://genetics.bwh.harvard.edu/pph/) where structural query options were set to default. A PSIC score above 0.85 identifies that the particular mutation was never or almost never observed in that protein family and would be classified as ‘probably-damaging’, scores of 0.15 to 0.85 classified as ‘possibly damaging’, and scores of <0.15 as ‘benign [70]. A second algorithm, SIFT (http://sift.jcvi.org/) was used to support the predicted effect of the *cauli* p.I855K amino acid substitution on protein function. A SIFT BLink analysis was performed using Ift140 protein ID NP_598887.3. Exonic splice enhancer analysis was performed using ESEfinder v3.0 (http://rulai.cshl.edu/cgi-bin/tools/ESE3/esefinder.cgi?process=home).

### Tissue collection

Adult female mice were anaesthetised with isoflurane and culled by cervical dislocation according to the National Health and Medical Research Council Australian code of practice for the care and use of animals for scientific purposes (RCH AEEC approval #A647). Embryos were dissected free from all extra embryonic membranes in Phosphate Buffered Saline (PBS) and processed dependent on the subsequent application. Between 2–6 independent embryos were processed for each analysis. Heterozygous embryos were indistinguishable to wild-type.

### Hematoxylin and Eosin (H&E) staining

Embryos were fixed in 4% PFA for up to several weeks then transferred to 70% Ethanol prior to processing through xylene and paraffin using an Excelsior ES tissue processor (Thermo Scientific). Tissues were embedded in the required orientation using a Histocentre 2 Embedding Station (Shandon Life Sciences) and 10 µM sections cut on an MR-2 microtome (RMC products). A standard H&E protocol was followed with 5 min staining in hematoxylin, 1 min staining in eosin and mounted with Entellan (Merck). Images were taken on a Nikon Eclipse 80i microscope (Pathtech).

### Skeletal preparations

Embryos >E16.5 were processed for skeletal preparations to visualize bone (Alizarin red) and cartilage (Alcian blue) as previously described [71], with modifications. All steps were carried out at room temperature. Embryos were skinned and eviscerated prior to fixation in 95% (v/v) ethanol. Once required, tissues were incubated in Alcian blue stain (0.06% (w/v) Alcian blue in 80% (v/v) ethanol, 20% (v/v) acetic acid) overnight, re-fixed in 95% ethanol for several hours and cleared in 2% (w/v) KOH×2 hr. Embryos were then incubated with Alizarin red solution (0.03% (w/v) Alizarin red in 1% (w/v) KOH) overnight then immersed in 1% (w/v) KOH/20% (v/v) glycerol solution for further clearing. For long-term storage, embryos were transferred into 50% (v/v) ethanol/50% (v/v) glycerol. Images were taken on a Leica MZ6 microscope using a Leica DFC290 camera (both Leica Microsystems Ltd).

### Scanning Electron Microscopy (SEM)

E10.5 limb buds were dissected from wild-type, heterozygous and homozygous embryos, fixed and processed for scanning electron microscopy as previously described [72]. Tissues were viewed using a Philips XL30 FE scanning electron microscope.

### *In situ* probe synthesis and whole mount hybridisation (WISH)

Total RNA was isolated from adult mouse brain tissue using a Qiagen RNeasy Midi Kit (Qiagen) or whole E11.5 wild-type embryos in Trizol (Invitrogen) by phenol/chloroform extraction. cDNA was generated from 1 µg RNA using random hexamers (Sigma) and BioScript reverse transcriptase (Bioline). cDNA fragments specific for the 3′UTR sequences of *myogenin*, *Sox9* and *Twist1* were amplified from RNA and ligated into pCRII-TOPO using the TOPO TA cloning kit (Invitrogen). Plasmids containing *Msx1*, *Shh*, *Ptch1*, *Grem1*, *Gli3*, *dHand*, *Dusp6* and *Fgf8* were obtained from C.Wicking, IMB, University of Queensland. RNA probes were generated by plasmid amplification with M13 primers, synthesised with T7 or SP6 RNA polymerase using a MAXIscript kit (Ambion), integrating dig-UTP (Roche). Probes were treated with Turbo DNase (Ambion) and unincorporated nucleotides removed using NucAway Spin Columns (Ambion). A sense probe was always generated for analysis of first-use probes.

E10.5–E13.5 *cauli* embryos were processed for whole mount *in situ* hybridisation as previously described [73], with modifications. After methanol rehydration, embryos were permeabilised by incubation in 10 µg/ml proteinase K for a time optimised for the stage of embryonic development; E10.5 for 45 min, increased per day of development by 10 min increments. Embryos were post-fixed in 0.2% glutaraldehyde/4% paraformaldehyde (PFA), equilibrated in prehybridisation buffer and incubated at 65°C overnight with the *in situ* riboprobe. After adequate washing to remove unbound probe, embryos were blocked with 10% sheep serum and incubated with an anti-digoxigenin-AP antibody (1∶1000, Roche). After additional washing, colour was developed with nitroblue tetrazolium (NBT) and 5-bromo-4-chloro-3-indolyl phosphate (BCIP) and the reaction stopped with EDTA buffer. The colour was fixed with 4% PFA, background staining reduced by storage in 100% methanol and images taken using a Nikon Eclipse 80i microscope (Pathtech).

### Immunohistochemistry

Embryos at E10.5 were processed for immunohistochemistry by fixation in 4% paraformaldehyde (PFA)×30 min, permeabilised/blocked in 0.1% Triton X-100/2% bovine serum albumin (BSA) in PBS, and washed for several hours/overnight in PBS. Embryos were incubated with a rabbit anti-Ift140 polyclonal antibody (1∶50; Proteintech) and mouse acetylated α-tubulin antibody (1∶2000; Sigma) overnight at 4°C, washed for several hrs/overnight in PBS and incubated overnight in the dark with Alexa Fluor 488-conjugated goat anti rabbit IgG and Alexa Fluor 594-conjugated goat anti mouse IgG secondary antibodies (both 1∶1500; Molecular Probes). Following several washes in PBS, embryos were post fixed in 4% PFA×5 min and re-washed in PBS. Embryo limb tips were dissected off and mounted with Vectasheild (Vector Laboratories).

For analysis of cellular architecture, E10.5 embryos were processed as above, incubated with a mouse monoclonal anti-E-cadherin (1∶200; BD Transduction Laboratories), secondary Alexa Fluor 594-conjugated goat anti mouse IgG secondary antibody (1∶1000; Molecular Probes) and incubated with Alexa Fluor 488 phalloidin (1∶1000; Molecular Probes).

Immunofluorescence was analysed using a Leica TCS SP2 laser scanning confocal microscope (Leica Microsystems).

### Immunoblotting and quantitative real-time RT-PCR

Independently, whole E11.5 wild-type, heterozygous and homozygous embryos were lysed by sonication (Vibra Cell, Sonics and Materials) in Laemlli SDS Sample Buffer plus 0.1 M DTT. A western blot was performed using standard procedures. Briefly, 100 µg of protein from each genotype was run through a 6 and 10% Tris-Glycine gel and transferred onto PDVF membrane (GE Healthcare) for 1 hour at 100 V. Membranes were preblocked with 5% non-fat milk and incubated with a rabbit anti-Ift140 polyclonal (1∶2000; Proteintech) or mouse anti-Human Transferrin Receptor monoclonal (1∶1000; Invitrogen) antibody. Protein was detected with rabbit (1∶2000; Cell Signaling Technologies) or mouse (1∶10,000; Dako) HRP-linked secondary antibodies, respectively. Blots were processed using the Amersham ELC Prime detection kit (VWR International). Results are representative of at least 3 independent immunoblots, with protein from a minimum of 2 mutant embryos. Protein levels were calculated using ImageQuant TL, v7 software (GE Healthcare Life Sciences).

Whole E13.5 embryos were collected in Trizol reagent (Invitrogen) and sonicated for RNA extraction. Total RNA was extracted from embryos of each genotype using standard chloroform extraction procedures. First-strand cDNA synthesis was performed on 1 µg RNA from each genotype using BioScript reverse transcriptase (BioLine) using random primers. Quantitative real-time PCR reactions were performed in triplicate on a Lightcycler 480 System (Roche) using GoTaq qPCR Master Mix (Promega). Data were normalised to the housekeeping gene TBP (TATA Binding Protein). Primer sequences are detailed in Table 1.

### Human patient samples

All human analyses were approved by the Royal Children's Hospital Human Research Ethics Committee project 31190. Genomic DNA was isolated from blood samples received from the proband (JS 1-1), his parents (JS 1-2 and JS 1-3) and saliva from the unaffected sibling (JS 1-4). The candidate gene *IFT140* (NM_014714.3) was sequenced, including all 31 exons, intron/exon boundaries and most of the 5′ and 3′ untranslated regions and any variations to the GRCh37 human reference assembly identified. Alterations in the nucleotide sequence of JS 1-1 were analysed using the following criteria: 1) segregation was consistent with recessive inheritance; 2) PCIS score >0.15 by Polyphen-2; 3) SNP allele frequency <0.005 in public databases dbSNP [74] and 1000 genomes [75]; 4) no evidence of homozygous mutant alleles in a cohort of normal healthy controls.
